# Versican is a potential therapeutic target in docetaxel-resistant prostate cancer

**DOI:** 10.18632/oncoscience.136

**Published:** 2015-03-02

**Authors:** Naoko Arichi, Yozo Mitsui, Miho Hiraki, Sigenobu Nakamura, Takeo Hiraoka, Masahiro Sumura, Hiroshi Hirata, Yuichiro Tanaka, Rajvir Dahiya, Hiroaki Yasumoto, Hiroaki Shiina

**Affiliations:** ^1^ Departments of Urology, Shimane University Faculty of Medicine, Izumo, Japan; ^2^ Department of Urology, San Francisco Veterans Affairs Medical Center and University of California San Francisco, San Francisco, California

**Keywords:** versican, taxane resistance, castration resistant prostate cancer, thalidomide chemotherapy

## Abstract

In the current study, we investigated a combination of docetaxel and thalidomide (DT therapy) in castration-resistant prostate cancer (CRPC) patients. We identified marker genes that predict the effect of DT therapy. Using an androgen-insensitive PC3 cell line, we established a docetaxel-resistant PC-3 cell line (DR-PC3). In DR-PC3 cells, DT therapy stronger inhibited proliferation/viability than docetaxel alone. Based on gene ontology analysis, we found versican as a selective gene. This result with the findings of cDNA microarray and validated by quantitative RT-PCR. In addition, the effect of DT therapy on cell viability was the same as the effect of docetaxel plus versican siRNA. In other words, silencing of versican can substitute for thalidomide. In the clinical setting, versican expression in prostate biopsy samples (before DT therapy) correlated with PSA reduction after DT therapy (p<0.05). Thus targeting versican is a potential therapeutic strategy in docetaxel-resistant prostate cancer.

## INTRODUCTION

Prostate cancer (PC) is one of the most common malignancies in the world, and the 2^nd^ leading cause of death among males in the US [1]. The recent application of PSA testing to routine clinical practice has led to more frequent detection of early and/or organ confined disease [2]. Despite this early or organ-confined detection of PC, over one-fourth of the patients with localized PC develop a biochemical recurrence (BCR) after radical surgery. Finally, PC with BCR is sure to progress towards the acquisition of castration-resistance [3]. Although recent advances in chemotherapeutic strategies against castration-resistant prostate cancer (CRPC) are likely to control its biological aggressiveness [4-6, 7], CRPC still harbors a significant amount of treatment difficulty with very few effective treatment strategies available.

Thalidomide is notorious for its potential toxicity during pregnancy, which leads to the malformation of the neonate organs [8]. However, recent advances in molecular biology have revealed a novel use of thalidomide as an inhibitor of angiogenesis through bFGF and VEGF pathways [9, 10]. Furthermore, another functional role of thalidomide as an essential regulator of inflammation-related cytokines has been postulated [11]. A recent publication has reported the potential benefit of clinical application of thalidomide through combined chemotherapy with docetaxel (DT therapy), which improved the prognosis of patients with CRPC [12, 13]. Interestingly, even in cases of docetaxel-resistant CRPC, the combination of thalidomide and docetaxel has shown a positive clinical response, suggesting the favorable reversal of docetaxel-resistance with second-line DT therapy [14].

In the present study, we investigated the rationale for DT therapy, especially the mechanism underlying the anti-cancer effect of thalidomide on taxane-resistant CRPC. For this purpose, we established docetaxel-resistant PC3 (DR-PC3) cells as an *in vitro* model of docetaxel-resistant, androgen-independent “prostate cancer” cells. In addition, using a cDNA microarray, we searched for reliable biomarkers that both parallel the biological effect of thalidomide on prostate cancer cells and predict better clinical outcomes when applying the DT therapy in docetaxel-resistant CRPC patients.

## RESULTS

### 1) Effect of single use of docetaxel, thalidomide, or combination of both on cell viability in the parent PC3 and DR-PC3 cells

**Figure 1:**
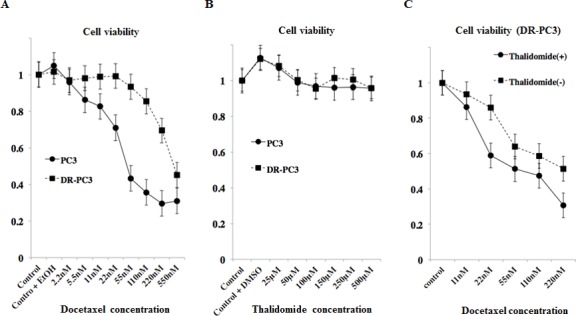
Cytotoxic effects of docetaxel, thalidomide, or combination of both on PC3 and DR-PC3 as shown by MTT assay A: The difference in cell viability between PC3 and DR-PC3 cells are shown in relation to docetaxel concentration. The cell viability of PC3 cells began to decrease at 2.2 nM of docetaxel, while viability of DR-PC3 was not reduced until 55 nM of docetaxel. *, P<0.05. ***, P<0.001. B: The cell viability of PC3 did not differ from that of DR-PC3, independent of thalidomide concentration. C: The cell viability of DR-PC3 cells treated with the combination of thalidomide (150 μM) and docetaxel was significantly lower than that of cells treated with docetaxel alone. *, P<0.05. **, P<0.01.

In DR-PC3 cells, the cell viability remained almost unchanged among the docetaxel concentrations of 2.2 nM and 55 nM, while higher docetaxel concentration of more than 110 nM resulted in a significant reduction of cell viability. In PC3 cells, even at the lower docetaxel concentration of 5.5 nM the cell viability was significantly reduced, and was decreased in a dose-dependent manner until 220 nM of docetaxel concentration (Fig. 1A). These results were compatible with the finding of established DR-PC3 cell lines harboring docetaxel-resistance. On the other hand, in both DR-PC3 and PC3 cells, the cell viability was not reduced, but kept unchanged in spite of increased concentration of thalidomide (Fig. 1B).

The effect of the combination of docetaxel with thalidomide on cell viability in DR-PC3 cells is shown in Figure 1C. Even in the lower concentration of docetaxel ranging from 11 nM to 220 nM, the cell viability was significantly lower in DR-PC3 cells treated with thalidomide (150 μM) than in those not treated with thalidomide, suggesting an additional and beneficial cytotoxic effect of thalidomide on DR-PC3 cells when simultaneously administered with lower doses of docetaxel.

### 2) cDNA microarray analysis to determine the candidate genes reflecting the thalidomide effect on DR-PC3 cells

**Figure 2:**
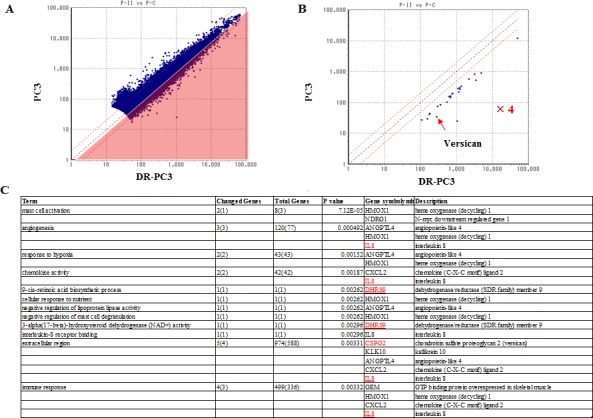
The difference in the gene expression between PC3 and DR-PC3 cells as shown by cDNA microarray results A: The area covered with light red corresponds to the genes which were increased by at least two-fold. Blue dots correspond to each gene analyzed. B: The genes with at least a four-fold increase in expression among DR-PC3 cells over PC3 cells are selectively shown. is indicated with a red arrow. C: The results of ontology analysis are shown. “Term” identifies the category of the ontology. The p value of statistical difference was based on the comparison made between the genes belonging to group A and the total series.

Using cDNA microarray, we identified 24 genes, which exhibited at least a four-fold increase in DR-PC3 compared with those in the parent PC3 (group A) (Fig. 2A, B) (Table.S1). Among these 24 genes, ontology analysis revealed that molecular function, cellular component and biological process were associated with 16 genes, 13 genes and 14 genes, respectively. The GO terms with a high degree of statistical difference between group A and the total series are shown in Figure 2C. Even if high statistical significance was found, a small number of genes changed and involved did hardly speculate a real significance. Based on this approach, the GO term corresponding to the extracellular region appeared to be more biologically relevant than the GO term, in which only few genes are involved. In this group A, angiogenesis (ANGPTL4, HMOX1, IL8), extracellular region (GSPG2, KLK10, ANGPTL4, CXCL2, IL8) and immune response (GEM, HMOX1, CXCL2, IL8) were significant GO terms. Multiple drug resistance genes were also found to be significant in the GO term, such as copper ion binding (MT1F and MT2A) and phosphoribulokinase activity (ABCB1) (data not shown).

**Figure 3:**
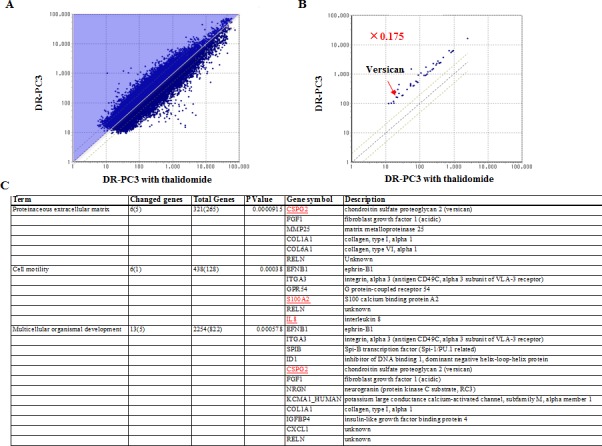
The difference in the gene expression between DR-PC3 cells with and without thalidomide treatment as shown by quantitative RT-PCR A: The light blue area includes the DR-PC3 cell genes with expression of less than 0.5-fold after thalidomide treatment when compared with no thalidomide treatment. B: The genes in DR-PC3 cells with less than 0.175-fold expression after thalidomide treatment compared to DR-PC3 cells without thalidomide treatment. The location of versican is indicated with a red arrow. C: The lower panel indicates the significant GO terms associated with decreased gene expression after thalidomide treatment in DRPC3 cells. The genes related to each GO term are depicted at right side of the panel.

We also found 47 genes, which were 0.175-fold decreased in DR-PC3 with thalidomide, compared with those in DR-PC3 without thalidomide treatment (Group B) (Fig. 3A, B) (Table.S2). Likewise, on the basis of ontology analysis, molecular function, cellular component and biological process were correlated with 35 genes, 34 genes and 31 genes, respectively. In this series, the biologically relevant GO terms were significantly associated with proteinaceous extracellular matrix, cell motility, and multicellular organismal development (Fig. 3C).

**Figure 4:**
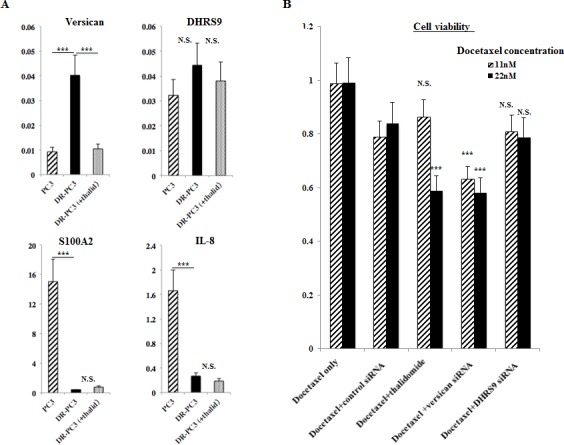
Effect of proposed target genes on cell viability of DR-PC3 cells as shown by gene-specific siRNA transfection A: Based on the cDNA microarray results, we selected versican, DHRS9, S100A2, and IL-8 genes. Among these four genes, only versican showed quantitative RT-PCR results that were compatible with the cDNA microarray analysis. ***, P<0.001. B: The cell viability of DRPC3 cells treated with docetaxel only, docetaxel with thalidomide, docetaxel with versican siRNA, or docetaxel with DHRS9 siRNA is shown. Cell viability was significantly lower in docetaxel with versican siRNA when compared to docetaxel with control siRNA at 11 and 22 nM of docetaxel. At 22 nM of docetaxel, the cell viability was not significantly different between docetaxel with thalidomide and docetaxel with versican siRNA. The effect of the combination of docetaxel with DHRS9 siRNA transfection on cell viability was minimal in comparison to the combination of docetaxel with versican siRNA transfection. ***, P<0.001.

We hypothesized that the genes common to groups A and B might be candidate genes which predict the biological effect of thalidomide on DR-PC3 cells. As shown in Figs 2 and 3, biologically relevant genes were selected and supposed to be versican, IL8, DHRS9 and S100A2. To validate the results obtained from cDNA microarray, we performed quantitative RT-PCR using primers for these four genes. As shown in Fig. 4A, expression of the versican mRNA transcript was significantly increased with acquisition of docetaxel-resistance and drastically decreased after thalidomide treatment. The pattern of DHRS9 expression was similar to that of versican; however, the difference in expression did not reach statistical significance. On the contrary, expression levels of S100A2 and IL-8 mRNA transcripts were not changed in the way that might be expected by the cDNA microarray. Thus, we selected versican and DHRS9 genes for further investigation into their involvement in docetaxel-resistance and thalidomide-induced reduction of cell viability.

### 3) Effect of siRNA transfection of versican and DHRS9 on cell viability in DR-PC3 cells with low-dose docetaxel treatment

Since the cytotoxic effect of lower dose docetaxel in combination with thalidomide on DR-PC3 cells was found to be significant (Fig. 1C), we investigated whether the transfection of versican or DHRS9 siRNA with low dose docetaxel might confer the same cytotoxic effect. As shown in Figure 4B, cell viability of DR-PC3 cells treated with versican siRNA and 22 nM of docetaxel was approximately 25% lower than that of cells treated with control siRNA and 22 nM of docetaxel, which difference in the cell viability was supposed to be the same as found in the difference between 22nM of docetaxel with and without thalidomide treatment (Fig. 1C). At 11 nM of docetaxel, cell viability was also significantly lower in docetaxel with versican siRNA when compared to docetaxel with control siRNA or docetaxel with thalidomide. In contrast, the difference in cell viability of DR-PC3 cells treated with DSHRS9 siRNA and 11 nM or 22 nM of docetaxel was not statistically different from those treated with control siRNA and 11 nM or 22 nM docetaxel (Fig. 4B). Thus, in the current *in vitro* study with low dose docetaxel, the effect of transfection of versican siRNA on DR-PC3 cells was comparable to thalidomide.

### Induction of apoptosis by combination of thalidomide with low dose docetaxel in DR-PC3 cells

**Figure 5:**
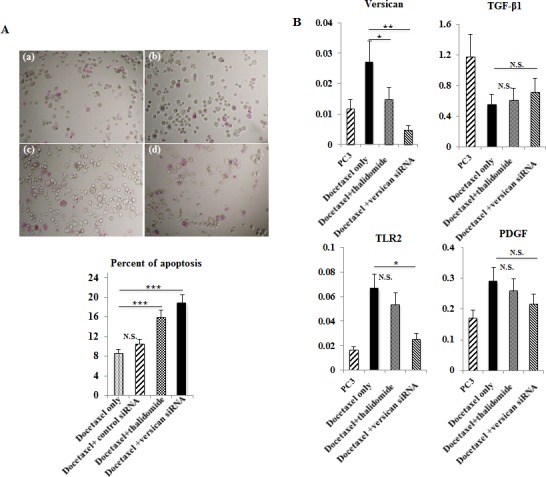
A: The DR-PC3 cells subject to apoptosis 48 hr after treatment The treatment of the DR-PC3 cells was 22 nM docetaxel only (a), 22 nM docetaxel with control siRNA transfection (b), 22 nM docetaxel with 250 mM thalidomide (c) and 22 nM docetaxel with versican siRNA transfection (d). The DR-PC3 cells stained with pink-dye are considered as positive for apoptosis. As compared with docetaxel only, there was a significant increase in apoptotic cells in docetaxel with thalidomide treatment or docetaxel with versican siRNA transfection. **, P<0.001. B: Gene expression of versican, TGF-â1, TLR2 and PDGF at mRNA transcript level as shown by quantitative RT-PCR. versican mRNA expression in DR-PC3 cells was significantly reduced in a stepwise manner from docetaxel alone, through docetaxel with thalidomide, and to docetaxel with versican siRNA transfection. Likewise, the alteration in TLR2 and PDGF mRNA expression was almost the same as that of versican ; however, the difference in the expression level between treatment groups was smaller for PDGF than TLR2. TGF-â1 expression did not show any significant difference between treatment groups. *, P<0.05.

As shown in Figure 5A, there was a significant increase in apoptotic cells in docetaxel with thalidomide treatment or docetaxel with versican siRNA transfection (p<0.001, p<0.001, respectively). However, no significant difference in the number of apoptotic cells was found between docetaxel with thalidomide treatment and docetaxel with versican siRNA transfection. As shown in Figure 5B, versican mRNA expression in DR-PC3 cells was significantly reduced in a stepwise manner from docetaxel alone, through docetaxel with thalidomide, and to docetaxel with versican siRNA transfection. Interestingly, TLR2 expression was also significantly decreased in a manner inverse to apoptotic cells in DR-PC3 cell cultures. On the other hand, while changes in PDGF mRNA expression exhibited the same pattern as that of TLR2, the differences did not reach statistical significance. Similarly, TGF-β1 expression did not show any significant difference between treatment groups.

### 5) Correlation of versican immunostaining with PSA reduction rate in docetaxel-resistant CRPC patients who received docetaxel in combination with thalidomide as second-line chemotherapy

**Figure 6:**
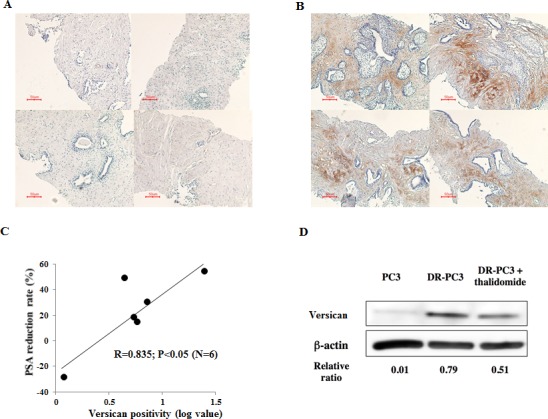
Correlation of versican immunostaining with PSA reduction rate in docetaxel-resistant CRPC patients who received docetaxel in combination with thalidomide A: Note that poor versican staining was found in the almost whole biopsy samples. B: Strong versican immunostaining was found in the prostate stroma near the cancer foci. C: versican immunostaining converted to log value was significantly correlated with PSA reduction rate in the 6 CRPC patients who underwent DT therapy after docetaxelresistance. Note that Case A showed negative versican immunostaining with poor PSA reduction rate, while Case B was positive for versican with better clinical response to DT therapy as determined by high PSA reduction rate. D: Western blotting of versican was analyzed using the protein samples of PC3, DR-PC3, and DR-PC3 treated with thalidomide. The relative intensity of versican to β-actin is shown in the “relative ratio”.

The PSA reduction rate was calculated between PSA values taken just before second-line chemotherapy and 1 month after initiation of chemotherapy. Typical immunostainings of versican with poor (A) or strong reactivity (B) are shown in Figure 6. The immunoreactivity of versican was not detected in PC cells, but was identified within the stromal component adjacent to the PC foci, suggesting that versican expression was controlled in a paracrine fashion. Although only 6 cases were analyzed in the current study, versican immunoreactivity converted to log value was significantly correlated with the PSA reduction rate, suggesting that versican immunoreactivity of the prostate-biopsy samples was predictive of the clinical response of the DT therapy. As exemplified by patient B in Figure 6B, docetaxel-resistant CRPC patients with strong expression of versican were more likely to respond to the chemotherapeutic strategy of docetaxel with thalidomide. As shown by Western blots, relative versican expression was significantly lower in DR-PC3 cells treated with docetaxel and thalidomide (0.51) than in those cells treated with docetaxel alone (0.79). Together, these data suggest that DT therapy can decrease versican expression and accelerate the apoptotic pathway in CRPC tissues.

## DISCUSSION

Despite the positive response to first-line docetaxel-based chemotherapy, re-application of docetaxel in combination with thalidomide (DT therapy) has been challenging for CRPC patients. In the case of taxane-resistant CRPC, the rationale for the usage of this second-line chemotherapeutic approach has not been well established and still remains to be elucidated. Recent publications have suggested a role for thalidomide in the transient reversal of the docetaxel-resistance in CRPC patients [14, 15].

First, we analyzed whether the effect of DT therapy on DR-PC3 cells might be biologically relevant as a second-line chemotherapeutic strategy. The cytotoxic potential of docetaxel could be reversed and/or accelerated with simultaneous thalidomide treatment even among castration-resistant PC cells that have acquired docetaxel-resistance. An *in vivo*, investigation has shown that thalidomide may contribute to the re-modeling of vascular structure, enabling docetaxel to better reach the cancer cells. However, it seemed unlikely that this was the explanation for the reversal of docetaxel-resistance with thalidomide in our current investigation of *in vitro* DR-PC3 cells. Thus, we sought to clarify the mechanism underlying this potential escape from docetaxel-resistance with thalidomide.

Again, limited numbers of chemotherapeutic strategies have been effective for and available to CRPC patients, especially second-line chemotherapies. DT therapy is a promising alternative [16, 17], however, docetaxel-resistance is not reversed in all CRPC patients treated with DT chemotherapy. Therefore, a biomarker or target gene that can reliably predict the clinical response to DT therapy must be determined. To identify such a biomarker, we screened for candidate genes that share the biologically binary characteristics of up-regulation after docetaxel-resistance and down-regulation following thalidomide treatment, or vice-versa. Based on the ontology analysis of the cDNA microarray results from DR-PC3 cells, we identified four genes fitting this description: versican, DHRS9, IL8, and S100A2. Quantitative RT-PCR revealed a pattern of mRNA expression that identified versican and DHRS9 as potential biomarkers worthy of further analysis. However, only siRNA for versican, not for DHRS9, reduced the cell viability of DR-PC3 cells when applied in combination with docetaxel. These findings provided a sufficient reason to focus on versican expression as a predictive marker of successful DT therapy in CRPC patients with docetaxel-resistance. As shown in Figure 6, the clinical response of DT therapy, as judged by the PSA reduction rate, was positively correlated with versican immunoreactivity, suggesting that CRPC patients with higher versican expression before DT therapy might be a best candidate for DT therapy. This is consistent with the correlation of versican with tumor initiation and/or progression in a variety of tumors [18-23], as well as the fact that prostate cancer cells have the ability to induce host stromal cells to accumulate versican via a paracrine mechanism [24, 25].

Next, we investigated whether the correlation of versican N mRNA transcript with cell viability after thalidomide treatment essentially reflected the biological function of versican gene or merely did serve as a secondary non-functional passerby without reflecting the cytotoxic effect of thalidomide on cell viability. The cell viability of DR-PC3 cells were significantly affected and reduced by the combination of low-dose docetaxel and versican siRNA transfection, suggesting that functional role of versican might be actively involved in the process of cell viability in the DR-PC3 cells. In addition, the DR-PC3 cells subject to apoptosis was significantly accelerated with simultaneous treatment of low-dose docetaxel after versican siRNA transfection, of which enhancement became almost the equal to what was induced by the combination of thalidomide with low-dose docetaxel. Either additional effect of thalidomide or inhibition of versican (versican siRNA transfection) on cell viability appeared to be compatible with each other in DR-PC3 treated with lower concentration of docetaxel, in turn indicating that thalidomide or versican siRNA has the probability of acting as a sensitizer of docetaxel.

In the present study, we found that versican immunostaining was increased in those CRPC patients with better PSA responses. The biological function of versican has previously been shown to generate a hospitable microenvironment for tumor invasion and metastasis. First, versican expression is up-regulated by TGF-β1 induced by PC cells in a paracrine fashion [26, 27]. Secondly, thalidomide is known to be a strong down-regulator of TGF-signaling [28]. In the current study, we could not verify the specific activation of TGF-β1 signaling. At the mRNA transcript level, TGF-β1 was reduced in DR-PC3 cells as compared to parent PC3 cells. Furthermore, there was no significant difference in the levels of TGF-β1 mRNA transcript found in DR-PC3 cells among the treatments of docetaxel only, docetaxel with thalidomide, and docetaxel with versican siRNA transfection. A more compatible explanation for the decreased expression of versican after thalidomide treatment is the inactivation of TNF-alpha [29]. A recent publication has clearly shown that through activation of TLR2 and TNF-alpha, versican can be accelerated to offer an appropriate environment for cancer cells to survive and pursue a metastatic spread [30]. In the current study, mRNA transcript of TLR2 was reduced after thalidomide treatment, running parallel with the down-regulation of versican expression. Additionally, the level of TLR2 mRNA transcript was decreased after transfection of versican siRNA, likely due to the decrease in the anti-apoptotic actions of versican on DR-PC3 cells. PDGF-B may also have a role in the action of thalidomide. PDGF-B signaling has an anti-angiogenic effect, similar to thalidomide, and it is also involved in the pathway of versican induction as constituents of TGF-β1 paracrine signaling [31]. We also found the stepwise reduction of PDGF expression in DR-PC3 cells after the combination of docetaxel with thalidomide or versican siRNA transfection as compared to docetaxel alone.

The effect of versican on the tumor environment appeared to provide the opportunity of tumor cells to get easily metastasis and invasion. Frequent events of versican over-expression in biologically active solid tumors have been reported [18-23], which appears to be in line with our results if increased expression of versican being common in docetaxel-resistant CRPC tissue. In vitro setting, the blockade of versican through versican siRNA transfection or thalidomide treatment could surely confer an apoptotic effect on DR-PC3 cells. Likewise, higher expression of versican was more likely to have a better PSA reduction rate. On the basis of these results, we can speculate that higher expression of versican means the higher possibility of having a best chance to draw the sufficient response coming from thalidomide treatment. We believe that the expression of versican just before the DT therapy in CRPC patients with docetaxel-resistance could be the best candidate biomarker for having the good response from DT therapy.

In an *in vitro* setting, we showed a sufficient cytotoxic effect of thalidomide with a combination of docetaxel even in the second round of chemotherapy, reducing cell viability by more than 25% as compared with thalidomide naïve PC3 cells. We believe this finding to be significant as these DR-PC3 cells share the biological characteristics of highly aggressive CRPC cells with the potential for multi-drug resistance as shown by cDNA microarray. *In vivo*, the additional role of thalidomide as a remodeler of the neovascularity around the cancer tissue may prove beneficial in delivering docetaxel to the PC environment. Thus, in the clinical setting, it is quite plausible that the anti-tumor effect of DT therapy might be accelerated. Besides, reduction of the cell viability after DT therapy was almost the same as that of versican siRNA transfection with docetaxel.

In conclusion, using gene ontology analysis focusing on the alteration of gene expression during the combination docetaxel with thalidomide in DR-PC3 cells, we have identified versican as a sophisticated biomarker for use in CRPC treatment. The changes of versican expression during DT therapy were well correlated with the clinical response of PSA reduction rate in docetaxel-resistant CRPC patients. Besides the clinical role of versican as a biomarker, the effect of versican siRNA transfection on docetaxel-resistant PC3 cells may be substituted for thalidomide. We believe that a novel strategy focusing on the inhibition of versican will provide a safe and effective alternative for treating patients with CRPC, especially with those who have acquired docetaxel-resistance.

## MATERIALS AND METHODS

### 1) Cell lines

Human androgen-independent prostate carcinoma cell line of PC was purchased from American Type Culture Collection (Rockville, MD, USA) and was maintained in RPMI 1640 medium containing 10% heat-inactivated fetal bovine serum (FBS) with 100 U/ml of penicillin and 100 mg/ml of streptomycin. docetaxel-resistant clones of PC3 were established by culturing the cells in docetaxel in a dose-escalation manner, according to the method described by Patterson *et al* [32]. After starting with the initial culture in 1 nM docetaxel, the surviving PC3 cells continued to divide through 4 passages under increased docetaxel concentration to a final concentration of 11 nM. Once PC3 cells were freely dividing in 11 nM docetaxel mediums, they were considered resistant against docetaxel and labeled as DR-PC3. All cell cultures were maintained at 70% confluency and medium was changed every 48 hr.

### 2) MTT assay

PC3 and DR-PC3 cells (5×10^5^) were plated in 96-well plates in RPMI-1640 containing 10% FBS at a final volume of 0.1 ml. The next day, the cells were treated with gene-specific siRNA or thalidomide, then MTT was added (20 ml/well of 5 g/L solution in PBS) after culturing for 24, 48, or 72 hr. After incubation at 37°C for 4 hr, the reaction was stopped by addition of 100 ml of DMSO. The reaction product was quantified by measuring absorbance at 490 nm using an ELISA reader (Wallac 1420 Victor 2, Victor Co., Finland) and HT-Soft software (Perkin-Elmer). All samples were assayed in 6 wells and repeated.

### 3) RNA extraction and cDNA microarray

Total RNA was extracted from PC3 or DR-PC3 cells with and without thalidomide treatment using TRIzol reagent (Invitrogen, Carlsbad, CA) and purified using the RNAsay system (Qiagen, Hilden, Germany). The RNA quality was determined in 1 M Tris-HCl (pH 7.5) using an Agilent 2100 bioanalyzer (Agilent Technology, Palo Alto, CA).

The microarray analysis was entrusted to Filgen, Inc. (Aichi, Japan). The microarray was conducted in two different series: one for the comparison of genes between parent PC3 cells and DR-PC3 cells, and the other for comparison between DR-PC3 cells under lower docetaxel concentration with and without thalidomide. The Human Genome Oligo Set V 4.0 arrays utilized herein consisted of 70-mer oligonucleotide probes (manufactured by Operon, Huntsville, AL), representing 34,394 genes. Prehybridization of the microarray was performed for 1 hr at 42 °C in a solution containing 5 × SSC (1×SSC is 0.15 M NaCl, 0.015 M sodium citrate), 0.1% sodium dodecyl sulfate (SDS), and 1% bovine serum albumin (BSA). The microarray was then washed at room temperature in distilled water, and dried by centrifugation at 200 g for 5 min.

Labeling of the aRNA was performed using 2 μg of each extracted total RNA using RNA Transcript SureLABEL Core Kit (Takara, Japan) and Cy5-UTP (Amersham Bioscience, NJ). The labeling aRNA of 3 μg was added to a solution containing 0.04 M Tris-acetate, 0.1 M potassium acetic acid, and 0.03 M magnesium acetic acid, and heated for 10 min at 94 °C. Hybridization for 16 hr at 42 °C was performed in 80 ml solution containing 5 × SSC, 0.1% × SDS, 10% formamide, and heat-denatured labeling aRNA. After hybridization, the microarray was washed at room temperature with 2 × SSC containing 0.1% × SDS for 4 min, once with 0.1 × SSC for 4 min, and three times with 0.1 × SSC for 1 min, and dried by centrifugation. The fluorescence images of Cy5 dye channels were obtained using a GenePix 4000B scanner (Axon Instruments, CA). Fluorescent hybridization signals of the microarray slide were analyzed with Array-Pro Analyzer ver 4.5 software (Media Cybernetics, Silver Spring, MD). The signal intensity of each spot and its local background were quantified, and net intensity was calculated by subtracting the background from the raw intensity. The database was analyzed using Microsoft Excel. The genes were categorized using the Microarray Data Analysis Tool ver 1.0 or 2.0 (supplied by Filgen, Inc.), based on the Gene-Ontology database http://geneontology.org/.

### 4) Quantitative RT-PCR

The total RNA extracted from the experimental cell cultures using Trizol was converted to cDNA by reverse trasnscriptase. Based on the cDNA microarray analysis, the common genes sharing the characteristics of up-regulation after docetaxel-resistance in PC3 cells and down-regulation in DR-PC3 cells under the combination of docetaxel with thalidomide were determined. The mRNA transcript level of each of the four genes selected from the microarray analysis–versican, DHRS9, IL-8 and S100A2– was measured by StepOne quantitative RT-PCR system (Applied BioSystem), in which GAPDH was used as a reference gene. The reaction protocol was strictly followed from the manufacturer's recommendations (Applied BioSystem). For each run, a standard curve was generated using a serial dilution of an external standard. The expression was calculated as the ratio of target gene to the reference GAPDH.

### 5) Treatment of DR-PC3 cells with small interfering RNA (siRNA) of versican DHRS9

Oligonucleotides representing siRNA against human versican expression, DHRS9 expression and mismatch control oligonucleotides were commercially purchased from Sigma for siRNA-versican and Ambion for siRNA-DHRS9 and siRNA-control. The sense strand for siRNA-versican and siRNA-DHRS9 is 5′-GAAAUCAACUCCCUGAUUATT-3′ and 5′-GGCAAUAAAUCCUAU GUGATT-3′, respectively. The anti-sense strand for versican and DHRS9 is 5′-UAAUCA GGGAGUUGAUUUCTT-3′ and 5′-UCACAUAGGAUUAUGCCTT-3′, respectively. For inhibition of versican, 10 mM siRNA oligonucleotides (siRNA-versican, siRNA-DHRS9 or siRNA-control) and 5 ml lipofectamine 2000 reagent (Invitrogen-Life Technologies Inc., Carlsbad, CA, USA) were diluted in 1 ml of Opti-MEMs I (Gibco, Carlsbad, CA, USA). Cells were transfected with lipofectamine + siRNA-versican or siRNA-DHRS9, lipofectamine + siRNA-control, or left untransfected. The transfection was terminated after 5hr by aspirating the transfection medium and adding fresh DMEM 1640 containing 10% FBS and 11 nM docetaxel. The non-adherent cells were washed off and the remaining cells were incubated in medium containing 11 nM docetaxel at 37 °C. Cells were harvested in TRIzol 48 hr after treatment for quantitative RT-PCR. Cells were lysed 72 hr after treatment for Western blot analysis to evaluate versican protein levels.

### 6) Apoptosis assay

DR-PC3 cells, which were treated with docetaxel only, docetaxel + control siRNA, docetaxel + thalidomide, and docetaxel + versican siRNA, were seeded in 96-well plates (3000 cells/well. After 48 hr, the culture medium was replaced with fresh RITC medium containing APOPercentage Dye Label (Biocolor Ltd., United Kingdom) and incubated for 1 hr at room temperature. Purple-red stained cells were identified as apoptotic cells. The number of purple-red stained cells/100 cells was determined.

### 7) Western blotting

Proteins (50 mg) were separated by SDS-PAGE on 3-8% Tris-Acetate gels or 4-12% Bis-Tris gels (NuPAGE1, Invitrogen), and transferred to PVDF membranes (Amersham Biosciences). Membranes were blocked with 5% non-fat milk and incubated with rabbit anti-human versican antibody (0.2 mg/ml, A4476, Sigma-Aldrich, St Louis, MO) at 4 °C overnight. Detection was performed with a peroxidase conjugated anti-rabbit secondary antibody and ECL-substrate (ECLTM Western blotting detection system, Amersham Biosciences). Membranes were exposed to chemiluminescence film (Amersham Biosciences), which was scanned into the computer for data analysis. Quantification of immunoreactive bands was performed using ImageJ (http://rsb.info.nih.gov/ij/).

### 8) Immunohistochemistry

Paraffin embedded materials of prostate biopsy samples obtained from 6 patients with docetaxel-resistant CRPC were used for immunostaining of versican. The biopsy samples were taken from transrectal ultrasonography (TRUS)-guided approach within 1 week of the administration of docetaxel and thalidomide. The PSA reduction rate was calculated using the PSA values obtained just before and 1 month after the DT chemotherapy. Immunostaining of versican was done on 5 μm-thick consecutive sections obtained from paraffin-embedded materials, using rabbit polyclonal antibody for versican (1:120; Transduction Laboratories, Lexington, KY). The slides were prepared with antigen retrieval using citrate buffer (10 mmol/L, pH 6.0) before incubation of primary antibody. In negative controls, the primary antibody was replaced with non-immune serum. 3,3′-Diaminobenzidine (Sigma-Aldrich) was used as the chromogen and counterstaining was done using hematoxylin. ImageJ was used to quantify the versican immunostaining, which was converted to log value. The correlation between PSA reduction rate and versican immunostaining converted to log value was examined. Written informed consent was obtained from each patient for molecular analysis of the resected specimen, and study was approved by the local ethnical committee of Shimane University Faculty of Medicine (20140919-4).

### 9) Statistical analysis

The difference in the value between 2 groups was statistically analyzed by an ANOVA test followed by a post-hoc test. The correlation of PSA reduction rate and logarithmic-converted versican immunoreactivity was analyzed using a Pearson's coefficient correlation. The results of cDNA microarray focusing on ontology analysis were statistically performed using the microarray data analysis tool supplied by the Filgen. P value of less than 0.05 was considered as statistically significant.

## SUPPLEMENTARY TABLES


