# Supplementary material for: Versican is a potential therapeutic target in docetaxel-resistant prostate cancer
Source: Oncoscience. 2015 Mar 2;2(2):193–204. doi: 10.18632/oncoscience.136 (PMC4381710; doi:10.18632/oncoscience.136)
Supplement: Supplementary file 1 [file oncoscience-02-0193-s001.pdf]

Table S1. 24 genes which exhibited at least a four-fold increase in DR-PC3 compared with those in the parent PC3

Hold change	Gene_symbol	Description
43.910669	ABCB1	Multidrug resistance protein 1 (P-glycoprotein 1)
8.754059	DHRS9	NADP-dependent retinol dehydrogenase/reductase
6.896527	IL8	Interleukin-8 precursor (IL-8)
5.787564	-	-
5.670966	ANGPTL4	Angiopoietin-related protein 4 precursor (Angiopoietin-like 4)
5.537209	NP_849143.1	-
5.526582	CXCL2	Macrophage inflammatory protein-2-alpha precursor (MIP2-alpha)
4.938293	GEM	GTP-binding protein GEM (GTP-binding mitogen-induced T-cell protein)
4.922103	-	-
4.496926	-	-
4.440964	-	-
4.439868	-	7 kDa protein
4.406438	CSPG2	Chondroitin sulfate proteoglycan core protein 2 (Versican)
4.304836	-	-
4.26661	ADAMTS1	ADAMTS-1 precursor
4.24635	PFKFB4	6-phosphofructo-2-kinase/fructose-2,6-biphosphatase 4
4.239998	MT2A	Metallothionein-II (MT-II)
4.186314	SDC2	Syndecan-2 precursor (Fibroglycan)
4.170903	KLK10	Kallikrein 10 precursor
4.158277	S100A2	S100 calcium-binding protein A2 (S-100L protein)
4.14429	-	-
4.039572	MT1F	Metallothionein-II (MT-II)
4.030795	HMOX1	Heme oxygenase 1
4.009836	NDRG1	NDRG1 protein (N-myc downstream regulated gene 1 protein)

Table S2. **47 genes which were 0.175-fold decreased in DR-PC3 with thalidomide, compared with those in DR-PC3 without thalidomide treatment**

Fold change	Gene_symbol	Description
0.029182	RELN	Reelin precursor
0.039699	SAT	Diamine acetyltransferase 1
0.05716	HAS2	Hyaluronan synthase 2
0.080649	DHRS9	NADP-dependent retinol dehydrogenase/reductase
0.082495	Q8IYS0_HUMAN	-
0.092826	IGFBP4	Insulin-like growth factor binding protein 4 precursor
0.101237	-	Non-protein coding transcript
0.104024	EFNB1	Ephrin-B1 precursor
0.11559	-	-
0.116186	NP_004684.1	cytokeratin type II
0.116728	OGT	UDP-N-acetylglucosamine--peptide N-acetylglucosaminyltransferase
0.122667	ID1	DNA-binding protein inhibitor ID-1
0.127164	Q8TBJ6_HUMAN	Alpha-N-acetylgalactosaminide alpha-2,6-sialyltransferase
0.128068	-	-
0.129315	PABPC5	Polyadenylate-binding protein 5
0.130578	FOXN4	Forkhead box protein N4
0.139907	IL8	Interleukin-8 precursor (IL-8)
0.140591	NP_849143.1	-
0.141159	ZNF385	Zinc finger protein 385
0.143008	Q9NT59_HUMAN	-
0.146223	ZYX	Zyxin (Zyxin 2)
0.146269	NUBP2	Nucleotide binding protein 2 (NBP 2)
0.147122	XP_291770.4	PREDICTED: similar to ankyrin repeat domain 30A; breast cancer antigen NY-BR-1
0.15004	PRSS33	protease, serine, 33
0.15432	EPN1_HUMAN	Epsin 1 (EPS-15 interacting protein 1)
0.154834	-	-
0.154963	KCMA1_HUMAN	Calcium-activated potassium channel alpha subunit 1
0.155249	TNFAIP3	Tumor necrosis factor, alpha-induced protein 3
0.155561	ITGA3	Integrin alpha-3 precursor (Galactoprotein B3)
0.157604	-	Hypothetical protein
0.160862	MMP25	Matrix metalloproteinase-25 precursor
0.161188	COL6A1	Collagen alpha 1(VI) chain precursor
0.165267	Q6PIE2_HUMAN	MGC9515 protein
0.166427	SOX7	Transcription factor SOX-7
0.167235	GPR54	KiSS-1 receptor (KiSS-1R)
0.169596	-	Hypothetical protein
0.170039	BTBD14B	transcriptional repressor NAC1
0.170741	COL1A1	Collagen alpha 1(I) chain precursor
0.170888	NRGN	Neurogranin (Ng)
0.170897	FGF1	Heparin-binding growth factor 1 precursor (HBGF-1)
0.171756	SPIB	Transcription factor Spi-B
0.172243	-	-
0.172304	S100A2	S100 calcium-binding protein A2 (S-100L protein)
0.172382	CXCL1	Growth regulated protein alpha precursor (CXCL1)
0.172852	NCS1_HUMAN	Neuronal calcium sensor 1 (NCS-1)
0.173317	CSPG2	Chondroitin sulfate proteoglycan core protein 2 (Versican)
0.174231	POFUT1	GDP-fucose protein O-fucosyltransferase 1 precursor
